# Long-term efficacy of high-frequency (10 kHz) spinal cord stimulation for the treatment of painful diabetic neuropathy: 24-Month results of a randomized controlled trial

**DOI:** 10.1016/j.diabres.2023.110865

**Published:** 2023-08-01

**Authors:** Erika A. Petersen, Thomas G. Stauss, James A. Scowcroft, Michael J. Jaasma, Elizabeth S. Brooks, Deborah R. Edgar, Judith L. White, Shawn M. Sills, Kasra Amirdelfan, Maged N. Guirguis, Jijun Xu, Cong Yu, Ali Nairizi, Denis G. Patterson, Kostandinos C. Tsoulfas, Michael J. Creamer, Vincent Galan, Richard H. Bundschu, Neel D. Mehta, Dawood Sayed, Shivanand P. Lad, David J. DiBenedetto, Khalid A. Sethi, Johnathan H. Goree, Matthew T. Bennett, Nathan J. Harrison, Atef F. Israel, Paul Chang, Paul W. Wu, Charles E. Argoff, Christian E. Nasr, Rod S. Taylor, David L. Caraway, Nagy A. Mekhail

**Affiliations:** a Department of Neurosurgery, University of Arkansas for Medical Sciences, 4301 W Markham St, Little Rock, AR 72205, USA; b Advanced Pain Management, 4131 W Loomis Rd Ste 300, Greenfield, WI 53221, USA; c Pain Management Associates, 200 NE Missouri Rd Ste 103, Lee’s Summit, MO 64086, USA; d Nevro Corp, 1800 Bridge Pkwy, Redwood City, CA 94065, USA; e Commexus Ltd, Dunblane, Scotland, UK; f AES Compass Orlando, 100 W Gore St, Orlando, FL 32806, USA; g Touchstone Interventional Pain Center, 2925 Siskiyou Blvd, Medford, OR 97504, USA; h IPM Medical Group, 450 N Wiget Ln, Walnut Creek, CA 94598, USA; i Ochsner Health System, 2820 Napoleon Ave, New Orleans, LA 70115, USA; j Department of Pain Management, Cleveland Clinic Foundation, 9500 Euclid Ave, Cleveland, OH 44195, USA; k Swedish Medical Center, 1101 Madison St, Seattle, WA 98104, USA; l Nevada Advanced Pain Specialists, 5578 Longley Ln, Reno, NV 89511, USA; m Central Florida Pain Relief Centers, 100 W Gore St #500, Orlando, FL 32806, USA; n Pain Care, 1365 Rock Quarry Rd #301, Stockbridge, GA 30281, USA; o Coastal Orthopedics and Sports Medicine, 8000 SR 64, Bradenton, FL 34212, USA; p Department of Anesthesiology, University of Arkansas for Medical Sciences, 4301 W Markham St, Little Rock, AR 72205, USA; q Department of Anesthesiology, Weill Cornell Medical College, 240 East 59th Street, 2nd Floor, New York, NY 10022, USA; r Department of Anesthesiology and Pain Medicine, University of Kansas Medical Center, 3901 Rainbow Blvd, Kansas City, KS 66160, USA; s Department of Neurosurgery, Duke University, 40 Duke Medicine Cir, Durham, NC 27710, USA; t Boston PainCare, 85 1st Ave, Waltham, MA 02451, USA; u Department of Neurosurgery, United Health Services, 46 Harrison St, Johnson City, NY 13790, USA; v Holy Cross Hospital, 5601 N Dixie Hwy #209, Fort Lauderdale, FL 33334, USA; w Department of Neurology, Albany Medical Center, 47 New Scotland Avenue, Albany, NY 12208, USA; x Division of Endocrinology, Department of Internal Medicine, The University of Arizona College of Medicine - Phoenix, 475 N 5th St, Phoenix, AZ 85004, USA; y MRC/CSO Social and Public Health Sciences Unit, Robertson Centre for Biostatistics, School of Health and Well Being, Clarice Pears Building, University of Glasgow, Glasgow, Scotland G12 8QQ, UK; z Evidence-Based Pain Management Research, Cleveland Clinic, 9500 Euclid Ave, Cleveland, OH 44195, USA

**Keywords:** Diabetes mellitus, Neuropathy, Spinal cord stimulation, Painful diabetic neuropathy, Sensory function

## Abstract

**Aims:**

To evaluate the long-term efficacy of high-frequency (10 kHz) spinal cord stimulation (SCS) for treating refractory painful diabetic neuropathy (PDN).

**Methods:**

The SENZA-PDN study was a prospective, multicenter, randomized controlled trial that compared conventional medical management (CMM) alone with 10 kHz SCS plus CMM (10 kHz SCS+CMM) in 216 patients with refractory PDN. After 6 months, participants with insufficient pain relief could cross over to the other treatment. In total, 142 patients with a 10 kHz SCS system were followed for 24 months, including 84 initial 10 kHz SCS+CMM recipients and 58 crossovers from CMM alone. Assessments included pain intensity, health-related quality of life (HRQoL), sleep, and neurological function. Investigators assessed neurological function via sensory, reflex, and motor tests. They identified a clinically meaningful improvement relative to the baseline assessment if there was a significant persistent improvement in neurological function that impacted the participant’s well-being and was attributable to a neurological finding.

**Results:**

At 24 months, 10 kHz SCS reduced pain by a mean of 79.9% compared to baseline, with 90.1% of participants experiencing ≥50% pain relief. Participants had significantly improved HRQoL and sleep, and 65.7% demonstrated clinically meaningful neurological improvement. Five (3.2%) SCS systems were explanted due to infection.

**Conclusions:**

Over 24 months, 10 kHz SCS provided durable pain relief and significant improvements in HRQoL and sleep. Furthermore, the majority of participants demonstrated neurological improvement. These long-term data support 10 kHz SCS as a safe and highly effective therapy for PDN.

Trial Registration: ClincalTrials.gov Identifier, NCT03228420.

## 1. Introduction

Globally, the number of people with diabetes has quadrupled in the last two decades [1]. Diabetic neuropathy (DN) is a frequent long-term complication of diabetes, with an estimated lifetime prevalence exceeding 50% [2]. Typically starting in the feet and gradually ascending to the lower legs [3], symptoms classically manifest as numbness, tingling/paresthesia, loss of protective sensation, impaired balance and weakness, and reduced response to stimuli such as cold temperature and pinprick [3]. Over time, as the feet become progressively insensate, the risk of injury increases, leading to a greater likelihood of falls, fractures, foot ulceration, and lower limb amputation [3–7].

Painful diabetic neuropathy (PDN), affecting up to 25% of people with diabetes [8], is characterized by painful burning, lancinating, tingling, and/or shooting sensations, which can be severe and continuous, particularly at night [3]. Consequently, individuals with PDN suffer significantly reduced health-related quality of life (HRQoL), impaired functionality, and comorbidities such as sleep disorders and depression/anxiety [9].

Long-term management of PDN usually includes the prescription of oral neuropathic pain medications [3]. However, the effectiveness of these medications is relatively low, as measured by the number needed to treat (NNT; the number of patients that need to be treated before we expect one extra patient to be a treatment responder versus the control treatment) [10–14]. Additionally, many patients discontinue these medications, primarily due to inadequate pain relief and/or intolerable side effects [15]. Although the FDA has recently approved an 8% capsaicin patch as an alternative treatment, its effectiveness is also limited [13,16]. Overall, existing pharmacotherapies fail to provide long-term pain relief for the majority of PDN patients.

Spinal cord stimulation (SCS) is a safe and reversible nonpharmacological therapy for chronic neuropathic pain. In the traditional form of SCS, low-frequency pulses (40–60 Hz) are applied to the spinal cord to induce continuous paresthesia over the painful area, thereby masking the pain sensation. A more recent SCS innovation uses high-frequency 10 kHz pulses (“10 kHz SCS”) to relieve pain without paresthesia, which may be more comfortable for patients [17,18]. Recent results from a large randomized controlled trial (RCT; “SENZA-PDN study”) show that 10 kHz SCS provides substantial pain relief in PDN patients, with many demonstrating improved sensation on neurological examination [19,20]. Based on these findings, the FDA approved 10 kHz SCS for this indication in 2021. The American Association of Clinical Endocrinology and experts in a recent Clinical Compendia Series from the American Diabetes Association have also recommended 10 kHz SCS therapy as a treatment for refractory PDN pain with a high level of evidence [3,21].

Given the severe and chronic nature of PDN, any analgesic treatment must provide robust, durable pain relief. Here, we present the results of the SENZA-PDN study after 6 and 24 months of treatment with 10 kHz SCS.

## 2. Materials and methods

### 2.1. Study design

The SENZA-PDN study was a multicenter, prospective, randomized, open-label clinical study conducted in the United States at 18 centers. A detailed description of the study design and procedures has previously been published [22]. Prior to study initiation, Institutional Review Board (IRB) approval was obtained (Western IRB or local site IRB, when applicable). The study was conducted following the Declaration of Helsinki and Good Clinical Practices, reported under the Consolidated Standards of Reporting Trials (CONSORT) guideline [19], and registered with ClinicalTrials.gov (NCT03228420).

### 2.2. Participants

To be eligible for study enrollment, patients had to have PDN symptoms for 12 months or longer that were refractory to current or prior treatment with a gabapentinoid and at least 1 other class of analgesic drug, experience lower limb pain intensity of ≥5 cm on a 10-cm visual analog scale (VAS), hemoglobin A1c (HbA1c) ≤10% (86 mmol/mol), and be suitable surgical candidates. Exclusion criteria included upper extremity pain intensity due to diabetic neuropathy of ≥3 cm on a 10-cm VAS, body mass index >45 kg/m^2^, daily opioid dosage >120 mg morphine equivalents, and not an appropriate candidate for implantation or study involvement based on a recent psychological assessment.

### 2.3. Randomization

Patients who met eligibility requirements and provided written informed consent were randomized (1:1) to conventional medical management (CMM) alone or 10 kHz SCS plus CMM (10 kHz SCS+CMM). Randomization procedures have previously been reported [19]. At the end of the 6-month randomized phase, patients could cross to the other treatment arm if they had less than 50% pain relief from baseline, were dissatisfied with their treatment, and the investigator agreed that the switch was appropriate. Patients who received 10 kHz SCS therapy (including the original 10 kHz SCS+CMM recipients and CMM-to-10 kHz SCS+CMM crossover patients) were followed up for 24 months postimplantation.

### 2.4. Interventions

Interventions have been described previously [19,20]. In brief, investigators administered CMM to all enrolled patients according to their standard of care for PDN and diabetes. Individuals receiving 10 kHz SCS underwent a 5–7 day trial with an external stimulator connected to 2 percutaneous leads placed in the posterior epidural space. Patients who experienced ≥50% pain relief from baseline (ie, trial success) were eligible to receive a permanent 10 kHz SCS system (Nevro Corp.) [17].

### 2.5. Outcomes

**Fig. 1.**
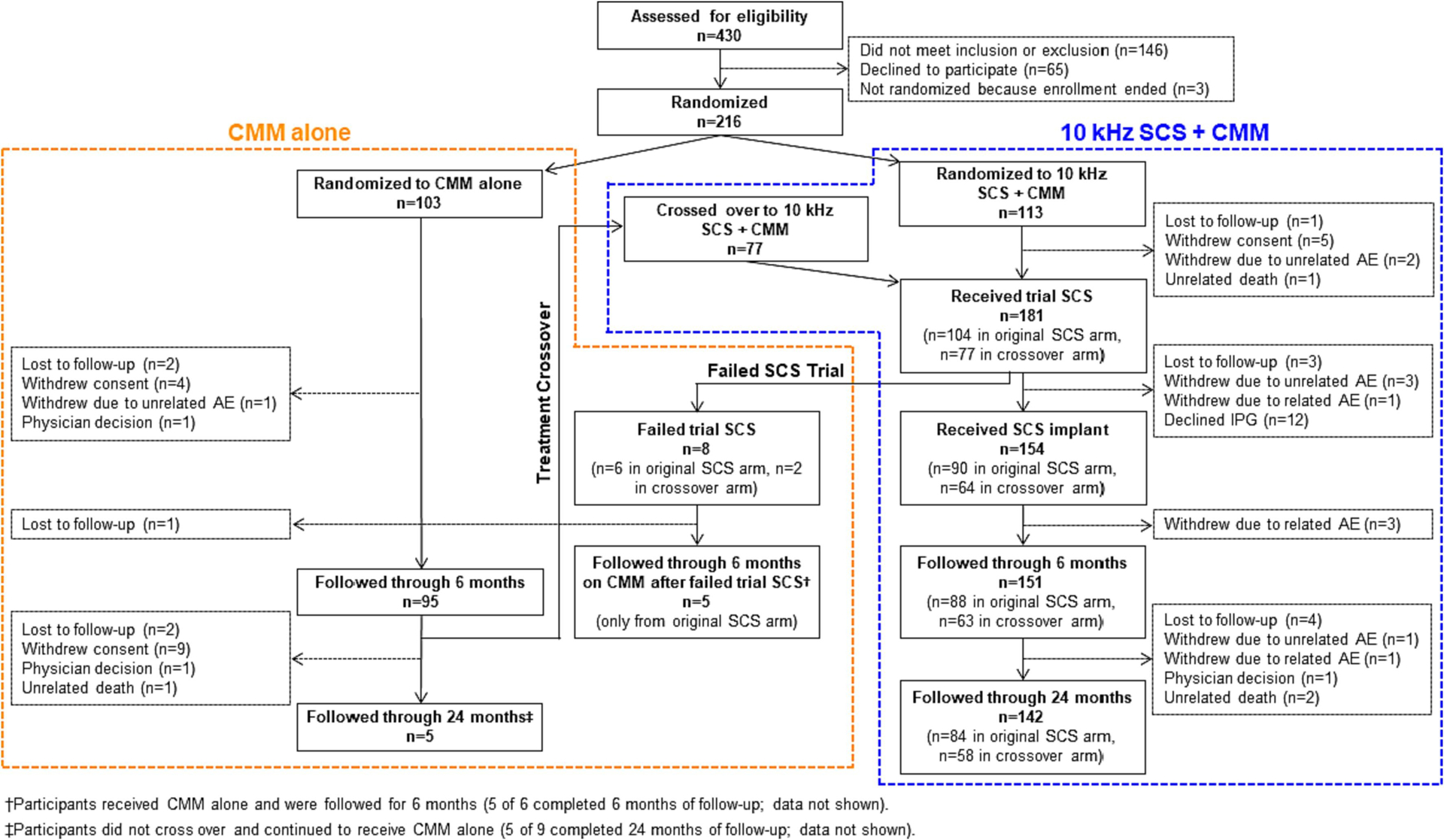
Disposition of All Screened Participants.

The investigators collected data at baseline, the end of the stimulation trial (all 10 kHz SCS+CMM recipients), and during regular follow-up intervals up to 24 months (Fig. 1). Assessments included measures of lower limb pain intensity (0–10 cm VAS) [23], patient-reported neuropathic pain (Douleur Neuropathique 4 Questions, DN4; 0–10 points scale) [24–26], pain interference with sleep (Pain and Sleep Questionnaire Three-Item Index, PSQ-3; 0–10 cm scale) [27], and HRQoL (EuroQol 5-Dimensional 5-Level questionnaire, EQ-5D-5L) [28], with HbA1c determined using standard blood tests.

Patient safety was also evaluated by monitoring adverse events and performing a comprehensive neurological evaluation at baseline and selected follow-up visits. As previously reported, independent neurologists developed this examination and trained the investigators to assess motor, sensory, and reflex function [19,20]. At each follow-up neurological examination, investigators used their clinical judgment to determine if there was a clinically meaningful deficit, no change, or a clinically meaningful improvement from baseline in each motor, sensory, and reflex function. From these results, overall neurological status was classified as improvement, maintenance, or deficit via the following criteria: improvement (improvement in at least 1 function without any deficit in any other function), maintenance (maintenance in all functions), or deficit (deficit in any function).

### 2.6. Statistical methods

A previous publication describes and reports the sample size calculation [22]. The current statistical analysis evaluated the results at the end of (1) the randomized phase, ie, 6 months after baseline, and (2) the postimplantation phase, ie, 24 months after implantation with a permanent 10 kHz SCS system (this includes the original 10 kHz SCS+CMM group and the CMM-to-10 kHz SCS+CMM crossover cohort). Statistical analyses were conducted separately for each phase.

For the randomized phase, results were evaluated for the CMM alone group and the 10 kHz SCS+CMM group. For the postimplantation phase, results were evaluated for (1) implanted participants from the original 10 kHz SCS+CMM group, (2) implanted participants from the CMM-to-10 kHz SCS+CMM crossover cohort, and (3) all implanted participants. We defined the preimplantation time point as study baseline in the original 10 kHz SCS+CMM group and the end of the randomized phase in the CMM-to-10 kHz SCS+CMM crossover patients.

In addition to raw scores and percentage change from baseline or preimplantation, we evaluated lower limb pain intensity VAS data at 6 and 24 months according to responders (≥50% pain relief from baseline or preimplantation) and profound responders (≥80% pain relief from baseline or preimplantation). Using standard methods, we also calculated the NNT [29].

Results are presented for all available data at each study visit. Statistical analyses were conducted in SAS (Version 9.4, SAS Institute Inc., Cary, NC). To statistically evaluate time and group effects for continuous and ordinal variables, missing data was first imputed via a multiple imputation procedure using all available nonmissing data for that outcome. Each imputed dataset was then analyzed with a repeated-measures linear model, and these results were summarized using the MIANALYZE procedure. The repeated-measures model included time and group as fixed effects and subject as a random effect, with an autoregressive correlation structure of order 1. Similar logistic and nonlinear repeated-measures models were used for proportions and percentage change in outcomes over time, respectively. However, imputation was not possible for 6 participants who withdrew prior to any neurological/sensory assessments follow-up. Consequently, these participants were classified as not having neurological or sensory improvement.

## 3. Results

### 3.1. Study population and demographics

The study investigators enrolled patients between August 28, 2017, and August 23, 2019 (Fig. 1). A total of 113 participants were randomized to receive 10 kHz SCS+CMM and 103 to CMM alone, with optional crossover at 6 months if specific criteria were met.

Baseline characteristics for the 216 randomized participants have previously been reported [19,20]. In brief, the participants had a mean age (SD) of 60.8 years (10.7), and 63% were male. Median durations (interquartile range) of diabetes and PDN symptoms were 10.9 (6.3–16.4) and 5.6 years (3.0–10.1), respectively. Baseline characteristics were comparable between the randomized groups. Baseline neurological exam results (reflexes, motor strength, and sensory function) were also similar between the randomized groups, with the only significant difference noted in a higher percentage of normal responses for light touch in the CMM alone group (73.9% vs 65.0% for the CMM alone and 10 kHz SCS+CMM groups, respectively; *P* <.001, Fisher’s Exact Test).

Among the 113 participants originally assigned to 10 kHz SCS+CMM, 104 completed the temporary stimulation trial, with 98 (94.2%) reporting pain relief of at least 50% from baseline (ie, trial success). Of the 90 patients who received a permanent system, 88 completed 6 months of follow-up, and 84 completed 24 months of follow-up. At 6 months, none of the original 10 kHz SCS+CMM recipients elected to cross over to the CMM alone arm.

In the control group (CMM alone), 95 participants completed the 6-month follow-up, with 83 eligible to cross over to the 10 kHz SCS+CMM arm. Of these, 77 (92.8%) crossed over and underwent a temporary stimulation trial, with 73 (94.8%) reporting trial success. Fifty-eight of the 64 patients who underwent permanent implantation completed 24 months of follow-up after their preimplantation assessment.

A total of 181 patients underwent a 10 kHz SCS trial, including the original 10 kHz SCS+CMM recipients and those who received 10 kHz SCS+CMM after crossing over from CMM alone, ie, the CMM-to-10 kHz SCS+CMM crossover group. Of these, 171 (94.5%) had a successful trial, 154 underwent permanent implantation, and 142 completed 24 months of follow-up (Fig. 1).

### 3.2. Safety

Through 24 months, no stimulation-related neurological deficits occurred, and no devices were explanted due to lack of efficacy. Among 154 permanently implanted participants, 7 (4.5%) experienced a study-related serious adverse event (SAE), and 8 (5.2%) had a procedure-related infection. Three infections resolved with standard treatment, while 5 required explantation (3.2% of all implanted patients). Of the 5 explanted patients, 4 exited the study, while 1 continued participation after reimplantation. One additional explant occurred as a precaution for an unrelated infection. Five other participants (3.2%) underwent revision surgery to reposition or replace the implantable pulse generator (IPG), and 3 (1.9%) had their leads repositioned or replaced due to migration. All participants with repositioned/replaced system components remained in the study. One additional infection occurred during the stimulation trial that resolved with standard treatment.

### 3.3. Lower limb pain relief

#### 3.3.1. Randomized phase

**Fig. 2.**
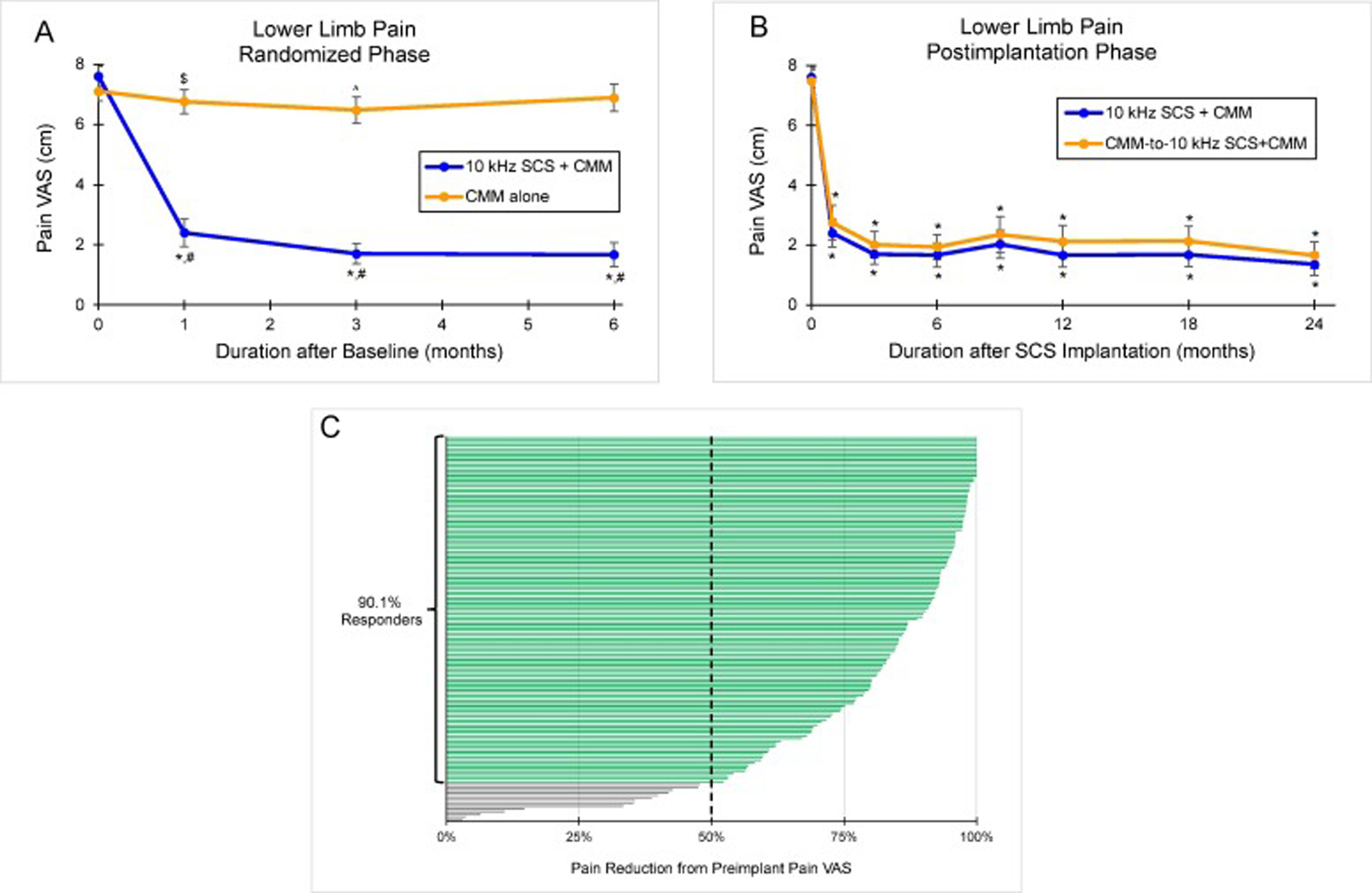
Lower Limb Pain Relief. Mean lower limb pain VAS scores (0–10 cm scale) during (A) the 6-month randomized phase and (B) the 24-month postimplantation phase. (C) Individual percentage pain reduction from preimplantation for all implanted participants who completed 24 months of follow-up: 90.1% (95% CI, 84.1%–94.0%; 128 of 142) were responders (≥50% pain reduction from preimplantation). Error bars indicate 95% CI; *P <.001 vs baseline or preimplantation; ^#^*P* <.001 vs CMM alone; ^$^*P* =.023 vs baseline; ^^^*P* =.003 vs baseline.

In the 10 kHz SCS recipients, lower limb pain scores assessed on a 10-cm visual analog scale (VAS) decreased by a mean of 76.4% (95% CI, 70.9%–81.9%; *P* <.001), from a baseline mean of 7.6 cm (95% CI, 7.3–7.9) to 1.7 cm (95% CI, 1.3–2.1; *P* <.001) at 6 months (Fig. 2A). In contrast, the control group had no significant change in lower limb pain VAS score, with a baseline mean of 7.1 cm (95% CI, 6.8–7.4) and a 6-month mean of 6.9 cm (95% CI, 6.4–7.3; *P* =.23 vs baseline; *P* <.001 vs 10 kHz SCS+CMM).

At 6 months, 85.2% (95% CI, 76.3%–91.2%; 75 of 88) of the 10 kHz SCS recipients were responders (≥50% pain relief from baseline), with 63.6% (95% CI, 53.2%–72.9%; 56 of 88) classified as profound responders (≥80% pain relief from baseline). In comparison, 6.3% (95% CI, 2.9%–13.1%; 6 of 95; *P* <.001 vs 10 kHz SCS+CMM) of the control group were responders, and 4.2% (95% CI, 1.6%–10.3%; 4 of 95; *P* <.001 vs 10 kHz SCS+CMM) were profound responders. The NNT for 50% pain relief at 6 months for 10 kHz SCS+CMM was 1.3 (95% CI, 1.2–1.5).

#### 3.3.2. Postimplantation phase

After 24 months of 10 kHz SCS, the mean lower limb pain VAS score in the group of all implanted patients decreased from a preimplantation mean of 7.6 cm (95% CI, 7.3–7.8) to 1.5 cm (95% CI, 1.2–1.8; *P* <.001), a mean reduction of 79.9% (95% CI, 76.3%–83.6%; *P* <.001). Pain relief and percentage pain relief at 24 months were consistent between the original 10 kHz SCS+CMM group and the CMM-to-10 kHz SCS+CMM crossover cohort (*P* =.22 for pain relief and *P* =.12 for percentage pain relief; Fig. 2B). At 24 months, 90.1% (95% CI, 84.1%–94.0%; 128 of 142) of the implanted patients were responders (Fig. 2C), with 65.5% (95% CI, 57.4%–72.8%; 93 of 142) classified as profound responders, and no patients had increased pain relative to baseline.

### 3.4. Patient-Reported neuropathic pain (DN4)

#### 3.4.1. Randomized phase

**Fig. 3.**
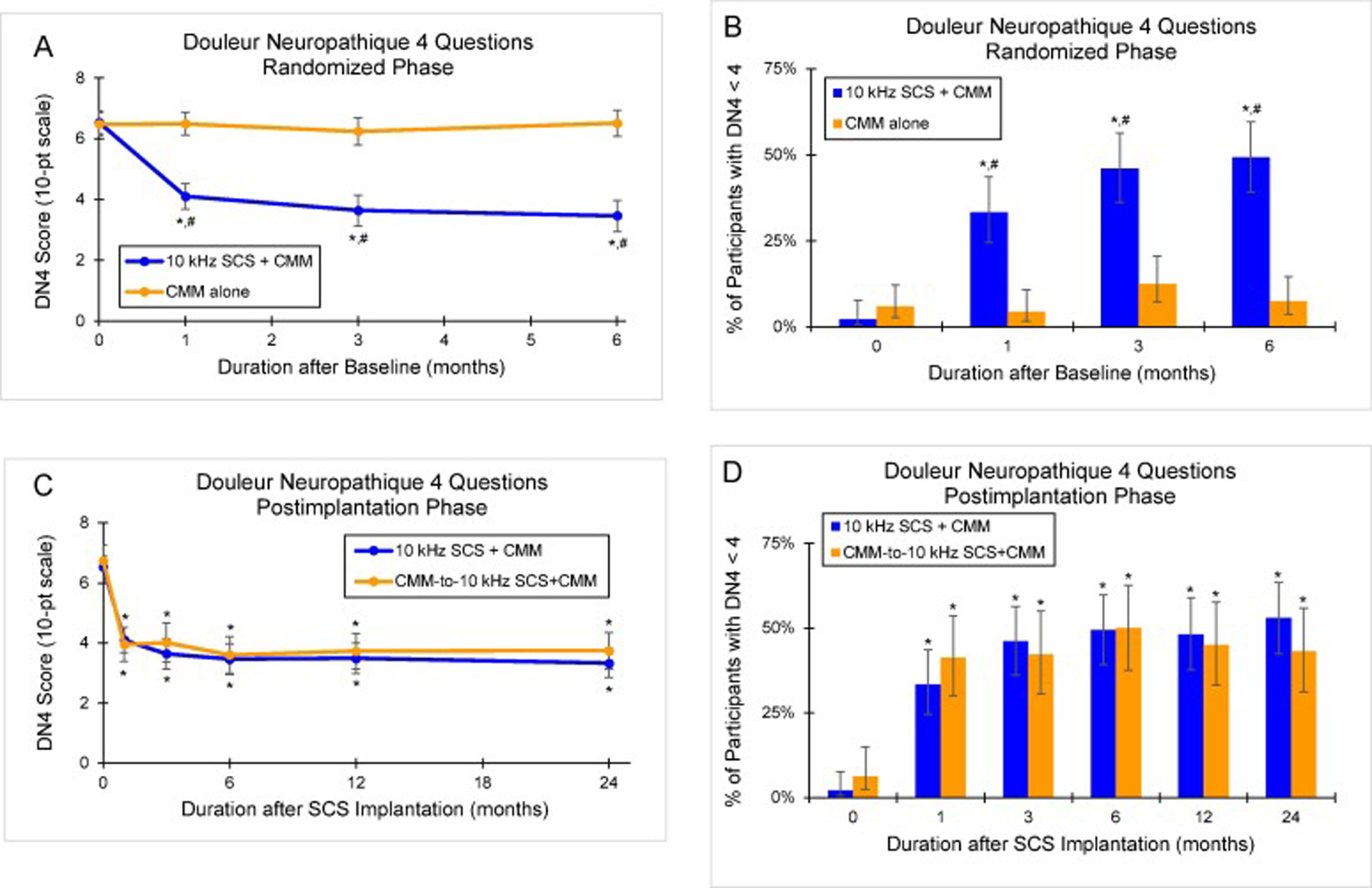
Patient-Reported Neuropathic Pain. The Douleur Neuropathique 4 Questions (DN4) scale is a validated neuropathic pain measure, with a score of ≥4 consistent with a clinical diagnosis of PDN. (A) Mean DN4 scores and (B) proportion of participants with DN4 <4 during the 6-month randomization phase. (C) Mean DN4 scores and (D) proportion of participants with DN4 <4 during the 24-month postimplantation phase. Error bars indicate 95% CI; *P <.001 vs baseline or preimplantation; ^#^*P* <.001 vs CMM alone.

During the randomized phase, the 10 kHz SCS recipients experienced a significant reduction in neuropathic pain, as measured by the Douleur Neuropathique questionnaire (DN4; 0–10 points scale). The mean DN4 score decreased from 6.5 (95% CI, 6.2–6.9) at baseline to 3.5 (95% CI, 3.0–4.0; *P* <.001) at 6 months, while control participants had no significant change (*P* =.79 vs baseline; *P* <.001 vs 10 kHz SCS+CMM; Fig. 3A).

A DN4 score ≥4 is consistent with clinically confirmed PDN. In the 10 kHz SCS recipients, the proportion with a DN4 score <4 increased significantly from 2.2% (95% CI, 0.6%–7.7%; 2 of 90) at baseline to 49.4% (95% CI, 39.2%–59.7%; 43 of 87; *P* <.001) at 6 months, while the corresponding proportion in the control group was unchanged (*P* =.34 vs baseline; *P* <.001 vs 10 kHz SCS+CMM; Fig. 3B).

#### 3.4.2. Postimplantation phase

Among all implanted patients, DN4 scores decreased from a preimplantation mean of 6.6 (95% CI, 6.3–6.9) to 3.5 (95% CI, 3.1–3.9; *P* <.001) after 24 months of 10 kHz SCS. This decrease in DN4 scores was due to a combination of reductions in the symptom subscore (mean reduction, 2.1; 95% CI, 1.7–2.4) and the examination findings subscore (mean reduction, 1.0; 95% CI, 0.8–1.3). In addition, the proportion of the group with a DN4 score <4 increased from 3.9% (95% CI, 1.8%–8.2%; 6 of 154) to 48.9% (95% CI, 40.8%–57.1%; 69 of 141; *P* <.001). At 24 months, DN4 results were comparable between the original 10 kHz SCS+CMM group and the CMM-to-10 kHz SCS+CMM crossover cohort (*P* =.14 for both outcomes; Fig. 3C and Fig. 3D).

### 3.5. Neurological outcomes

#### 3.5.1. Randomized phase

**Fig. 4.**
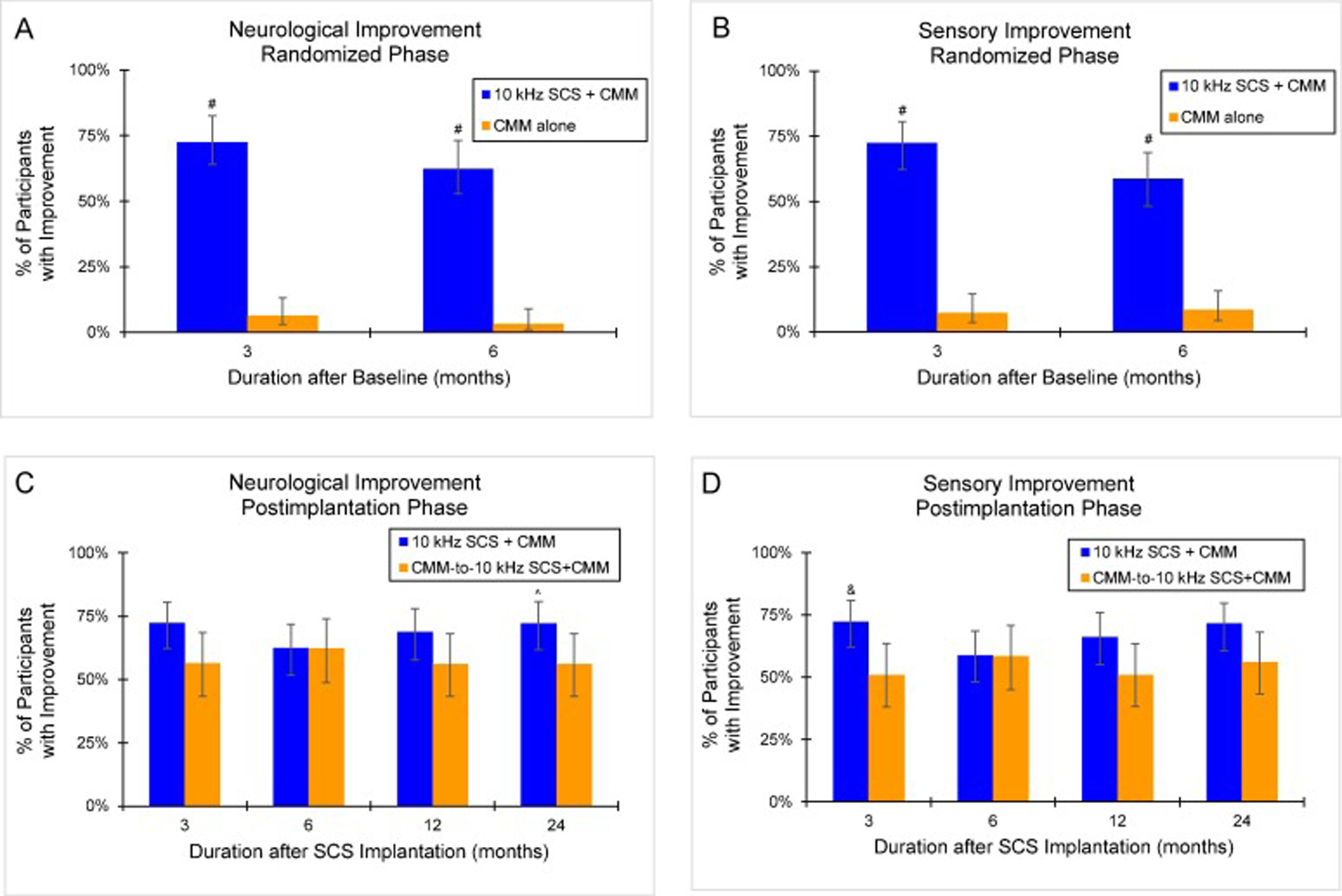
Neurological Outcomes. Standardized neurological assessments were performed at baseline and follow-up visits, including motor, sensory, and reflex tests. (A) Proportion of participants with a clinically meaningful improvement in sensory, motor, or reflex function from study baseline (without deficit in any category) during the 6-month randomized phase. (B) Proportion of participants with a clinically meaningful improvement in sensory function from study baseline during the 6-month randomized phase. (C) Proportion of participants with a clinically meaningful improvement in sensory, motor, or reflex function from study baseline (without deficit in any category) during the 24-month postimplantation phase. (D) Proportion of participants with a clinically meaningful improvement in sensory function from study baseline during the 24-month postimplantation phase. Error bars indicate 95% CI; ^#^*P* <.001 vs CMM alone; ^*P* =.048 vs CMM crossover to 10 kHz SCS+CMM; &*P* =.021 vs CMM crossover to 10 kHz SCS+CMM.

At 6 months, significantly more 10 kHz SCS recipients demonstrated a clinically meaningful improvement in sensory, motor, or reflex function from study baseline without deterioration in any category (62.4% of participants assessed to have improved; 95% CI, 51.7%–71.9%; 53 of 85) compared to the control group (3.2% of participants assessed to have improved; 95% CI, 1.1%–9.0%; 3 of 94; *P* <.001 vs 10 kHz SCS+CMM; Fig. 4A). Most of the improvements noted with 10 kHz SCS were in sensory function (58.8% of participants assessed to have improved; 95% CI, 48.2%–68.7%; 50 of 85; *P* <.001 vs CMM alone; Fig. 4B).

#### 3.5.2. Postimplantation phase

Investigators assessed neurological function versus study baseline in all implanted patients. After 24 months of 10 kHz SCS, 92 of 140 implanted individuals (65.7%; 95% CI, 57.5%–73.1%) exhibited a clinically meaningful improvement over study baseline in sensory, motor, or reflex function, without worsening in any category. Most of the neurological gains were observed in sensory function (65.0% of participants assessed to have improved; 95% CI, 56.8%–72.4%; 91 of 140). Additionally, the reported neurological and sensory improvement outcomes were similar between the original 10 kHz SCS+CMM group and the CMM-to-10 kHz SCS+CMM crossover cohort, with the initial 10 kHz SCS recipients showing higher improvement rates that reached statistical significance for neurological function at 24 months postimplantation (*P* =.048 for neurological improvement and *P* =.076 for sensory improvement; Fig. 4C and Fig. 4D).

### 3.6. Health-Related quality of life

#### 3.6.1. Randomized phase

**Fig. 5.**
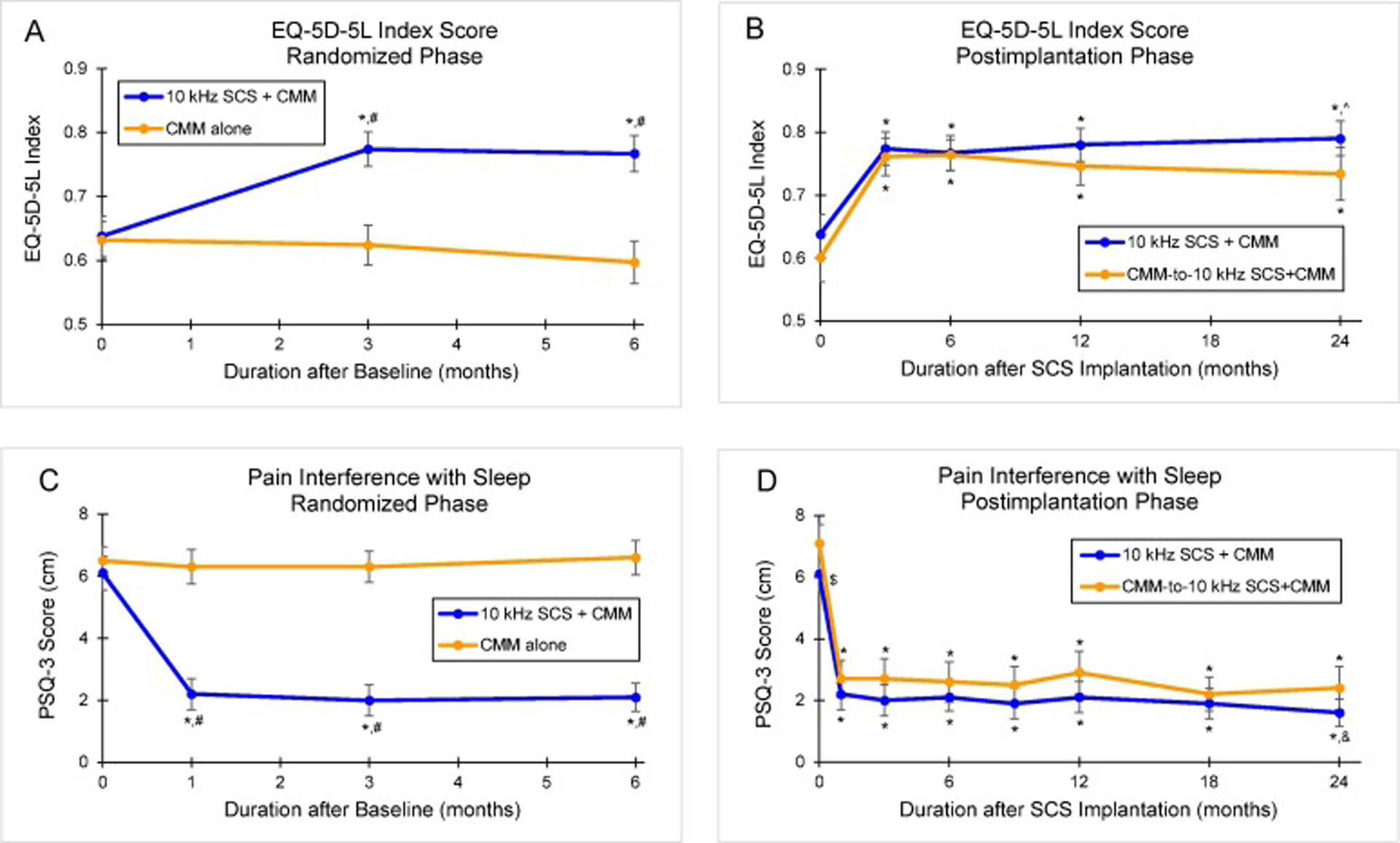
Health-Related Quality of Life (HRQoL) and Pain Interference with Sleep. The EuroQol 5-Dimensional 5-Level Questionnaire (EQ-5D-5L) measures HRQoL. The Pain and Sleep Questionnaire Three-Item Index (PSQ-3) assesses how often pain interferes with sleep (0-never; 10-always). Mean EQ-5D-5L index values during (A) the 6-month randomized phase and (B) the 24-month postimplantation phase. Mean PSQ-3 scores during (C) the 6-month randomized phase and (D) the 24-month postimplantation phase. Error bars indicate 95% CI; *P <.001 vs baseline or preimplantation; ^#^*P* <.001 vs CMM alone; ^*P* =.006 vs CMM crossover to 10 kHz SCS+CMM; ^$^*P* =.012 vs CMM crossover to 10 kHz SCS+CMM; ^&^*P* =.020 vs CMM crossover to 10 kHz SCS+CMM.

Health-related quality of life significantly improved after 6 months of 10 kHz SCS, based on the EuroQol 5-Dimensional 5-Level (EQ-5D-5L) questionnaire. The mean EQ-5D-5L index value increased by 0.130 (95% CI, 0.097–0.163; *P* <.001) from baseline in the 10 kHz SCS recipients, while the index value for the control participants declined by 0.031 but was not significantly different from baseline (*P* =.090; Fig. 5A). Additionally, at the 6-month assessment, the 10 kHz SCS recipients had significantly higher HRQoL than the control participants (*P* <.001).

#### 3.6.2. Postimplantation phase

Among all implanted patients, the mean EQ-5D-5L index value increased by 0.146 (95% CI, 0.117–0.175; *P* <.001) from preimplantation to 24 months, with the improvement in HRQoL consistent between the original 10 kHz SCS+CMM group and the CMM-to-10 kHz SCS+CMM crossover cohort (*P* =.37; Fig. 5B).

### 3.7. Pain interference with sleep

#### 3.7.1. Randomized phase

Baseline sleep quality was poor among all study participants, as shown by mean scores on the Pain and Sleep Questionnaire 3-Item Index (PSQ-3; 0–10 cm scale) of 6.1 cm (95% CI, 5.5–6.6) in the 10 kHz SCS recipients and 6.5 cm (95% CI, 6.0–6.9) in the control group. Pain interference with sleep reduced by a mean of 62.1% (95% CI, 54.6%–69.7%; *P* <.001) after 6 months of 10 kHz SCS, resulting in a mean PSQ-3 score of 2.1 cm (95% CI, 1.7–2.6), while control participants had no significant change (*P* =.91 vs baseline; *P* <.001 vs 10 kHz SCS+CMM; Fig. 5C).

#### 3.7.2. Postimplantation phase

Treatment with 10 kHz SCS significantly reduced pain interference with sleep in the group of all implanted patients over 24 months, as demonstrated by a 65.5% (95% CI, 57.5%–73.5%; *P* <.001) decrease in the mean PSQ-3 score, from 6.5 cm (95% CI, 6.1–6.9) at preimplantation to 1.9 cm (95% CI, 1.6–2.3; *P* <.001) at 24 months. In addition, the improvement in sleep quality was comparable between the original 10 kHz SCS+CMM group and the CMM-to-10 kHz SCS+CMM crossover cohort (*P* =.93; Fig. 5D).

### 3.8. Glycemic control

#### 3.8.1. Randomized phase

The 10 kHz SCS recipients had a mean HbA1c of 7.4% (95% CI, 7.1–7.6) (mean, 57 mmol/mol; 95% CI, 54–60) at baseline and 7.5% (95% CI, 7.2–7.8; *P* =.51) (mean, 59 mmol/mol; 95% CI, 55–62) after 6 months of 10 kHz SCS. In the control group, mean HbA1c was 7.4% (95% CI, 7.2–7.7) (mean, 57 mmol/mol; 95% CI, 55–61) at baseline and 7.5% (95% CI, 7.2–7.8; *P* =.45) (mean, 59 mmol/mol; 95% CI, 55–62) at 6 months.

#### 3.8.2. Postimplantation phase

In the group of all implanted patients, mean HbA1c was 7.5% (95% CI, 7.3–7.7) (mean, 59 mmol/mol; 95% CI, 56–61) at preimplantation and 7.3% (95% CI, 7.1–7.6; *P* =.11) (mean, 56 mmol/mol; 95% CI, 54–60) after 24 months of 10 kHz SCS. The mean percentage change over 24 months was similar between the original 10 kHz SCS+CMM group and the CMM-to-10 kHz SCS+CMM crossover cohort (*P* =.99).

## 4. Discussion

The SENZA-PDN study, the largest RCT to evaluate SCS in PDN patients to date, found that 10 kHz SCS provided significant and durable pain relief as well as improved HRQoL and sleep over the 24-month study period. Comparable to the 6- and 12-month study outcomes, the 24-month results confirm the robust and long-lasting beneficial effects of 10 kHz SCS in PDN patients [19,20,30]. These are important findings, given the severe, chronic, and debilitating nature of PDN and the lack of effective treatment options for this patient population. Moreover, investigators observed neurological improvement in a remarkably high proportion of patients after 10 kHz SCS treatment. To our knowledge, no other SCS treatment for PDN has exhibited this potentially disease-modifying effect. For each cohort evaluated, HbA1c did not show a statistically significant change over the 24-month follow-up period, supporting that the study results were not due to changes in glycemic control.

### 4.1. 10 kHz SCS versus traditional low-frequency SCS

Compared to previous RCTs that evaluated traditional paresthesia-based low-frequency SCS (LF-SCS) in similar PDN patients with a similar study design and control treatment [31,32], 10 kHz SCS treatment resulted in a greater reduction in pain at 6 months than LF-SCS (76% vs 41%–55%, respectively) and a higher responder rate for 50% pain relief (85% vs 53%–69%, respectively). By 24 months, the differences in pain reduction (80% vs 39%, respectively) and responder rate (90% vs 41%, respectively) were even more pronounced [33]. Evidence from a 6-month indirect comparison of RCT stimulation arm outcomes in PDN supports this observation, with the analysis finding significantly higher pain relief and responder rate for 10 kHz SCS over LF-SCS [18].

In the long-term LF-SCS RCT, increasing pain scores between 6 and 24 months suggest diminishing pain relief over time [34], a phenomenon observed in other chronic pain indications treated with LF-SCS [35–41]. According to the published literature, loss of efficacy during LF-SCS affects 10% to 25% of patients [35,38,41], accounts for 40% to 75% of all explants [35,42–45], and often occurs within 1 year [35]. In contrast, the high level of pain relief with 10 kHz SCS in the current study was durable through 24 months. Moreover, 10 kHz SCS provides pain relief without paresthesia, which may be more comfortable and tolerable than LF-SCS, especially in PDN patients who experience severe disease-induced paresthesia.

In the same long-term LF-SCS RCT, several HRQoL and sleep parameters did not differ from CMM at 6 months and did not show significant improvement over baseline at 24 months [32,34]. In comparison, patients who received 10 kHz SCS had significantly better HRQoL and sleep at 6 months than those treated with CMM, and these benefits lasted for the full 24 months. In particular, the increase of 0.146 in the mean EQ-5D-5L index value with 10 kHz SCS is highly clinically relevant, based on an estimated minimally important difference (MID) of 0.03 to 0.05 in patients with type 2 diabetes mellitus (ie, 2.9 to 4.9 times the MID) [46]. The long-term improvement in sleep with 10 kHz SCS is also important because patients with chronic pain repeatedly indicate that sleep is a high-priority treatment outcome [47–49].

### 4.2. 10 kHz SCS versus pharmacotherapy

The effectiveness of 10 kHz SCS compared to pharmacotherapy also appears very favorable. The NNT values for duloxetine, pregabalin, and gabapentin in PDN and neuropathic pain (including PDN pain) for the outcome of ≥50% pain relief versus placebo are reported to range from 5 to 16 at follow-up of 16 weeks or less [10–13]. Tricyclic antidepressants, mainly amitriptyline, appear to be more effective (NNT, 3.6); however, amitriptyline is often poorly tolerated [13].

In contrast to pharmacotherapy, the SENZA-PDN study had very low NNT values for ≥50% pain relief versus CMM alone at 1, 3, and 6 months, with values of 1.4, 1.2, and 1.3, respectively. These NNT values indicate that PDN patients who are suitable candidates for 10 kHz SCS have a much higher likelihood of treatment success than with CMM alone. Of course, it should be kept in mind when reviewing the NNT values that the control arms in the SENZA-PDN study and the pharmacotherapy trials were not identical, even if the overall study populations, timeframe, and outcome measures were similar.

### 4.3. Safety

The incidence and type of procedure-related complications were comparable to those reported in the SCS literature for all patient populations [50]. SAE rates were very low, and lead migration, IPG/lead revision surgery, and explant rates were at the lower end of the reported ranges for SCS [50]. Considering that patients with diabetes can be more susceptible to infection, it was reassuring to find that the incidence of infection during the SENZA-PDN study (5.2%) was within the range for SCS in other indications (3.4%–10.0%; mean, 5.2%; excluding PDN studies) [50]. Furthermore, the rate of explant due to infection in the current study was low, at 3.2%, and no devices were explanted due to lack of efficacy. Overall, the safety results support that patients with diabetes have no additional risk of complications with SCS.

### 4.4. Neurological/Sensory improvement

The neurological benefits observed after treatment with 10 kHz SCS are another noteworthy finding. Relative to their baseline examination, 66% of the patients had improved neurological status at the 24-month assessment, with improvement most often in the sensory domain. A recent small, open-label clinical study demonstrated statistically significant increases in intraepidermal nerve fiber density in the lower limb after 6 and 12 months of 10 kHz SCS [51], providing a potential mechanism for the observed improvements in sensory function. Interestingly, participants with sensory improvement at 24 months had higher preimplantation HbA1c compared to those with no change or reduced sensory function at 24 months (HbA1c, 7.7% vs. 7.2%; *P* =.021 via *t*-test), which suggests that patients with higher preimplantation HbA1c may be more likely to experience sensory improvement with 10 kHz SCS. These outcomes merit further exploration, given the increased risk of foot ulceration and lower limb amputation associated with diabetic neuropathy [6,7]. Based on our review of the literature, we are unaware of other SCS therapies associated with this potentially disease-modifying effect of 10 kHz SCS [52].

### 4.5. Study limitations

This pragmatic study aimed to provide high-level evidence to aid clinical decision-making, albeit with some limitations. Comparing an implanted SCS device with CMM made it impossible to blind participants and study personnel, leading to a risk of biased outcomes and a possible placebo effect. However, the results through 24 months with 10 kHz SCS were consistent, indicating minimal placebo effect.

Another limitation is the potential impact of missed visits on study results. However, relatively few visits were missed despite conducting the study during the COVID-19 pandemic. Furthermore, we found minimal impact across study outcomes when we accounted for missing data using a multiple imputation method. For example, the mean reduction in pain was 79.9% among all implanted participants at 24 months using all available data and 79.7% using all available data plus imputed data. The corresponding responder rates were 90.1% and 89.5%, respectively.

When interpreting the observed neurological improvements in this study, it is important to acknowledge certain limitations. Firstly, although the examination was developed as part of an RCT, it was not based on a validated scale. Additionally, the assessments of neurological status were conducted by trained clinicians who relied on their clinical judgment to determine changes. Despite standardized training, there is a possibility that inter-assessor variability may have influenced the test results. Furthermore, it should be noted that the investigators were not blinded to the treatment allocation when evaluating the neurological outcomes. Lastly, the assessment of neurological outcomes was performed relative to the study *baseline* examination for all patients in the study. Specifically, the 24-month postimplantation assessment in the CMM-to-10 kHz SCS+CMM crossover participants corresponded to 30 months after the study baseline exam, while the original 10 kHz SCS recipients were evaluated at 24 months after the study baseline exam. It is possible that the crossover cohort clinically deteriorated during the extra 6 months of CMM-only treatment prior to implantation, which may have contributed to their lower overall neurological and sensory improvements and the between-group difference in neurological outcome that achieved statistical significance at 24 months (*P* =.048, original 10 kHz SCS recipients vs CMM-to-SCS+CMM crossover group). While both groups still had meaningful neurological improvements, these outcomes suggest that delaying treatment for indicated patients could result in further neurological decline over time.

## 5. Conclusions

The results of this randomized controlled trial demonstrate robust, high-level evidence that 10 kHz SCS provides significant and durable pain relief in patients with refractory lower limb PDN pain. In addition, 10 kHz SCS treatment resulted in significantly improved sleep and HRQoL at 24 months, as well as neurological improvement in the majority of patients, providing a comprehensive solution for PDN management. The remarkable 24-month responder rate of 90% demonstrates that the therapy is highly effective and an important nonpharmacological therapy option. Furthermore, consistent pain relief for the original recipients of 10 kHz SCS and the implanted crossover participants (who received 10 kHz SCS after 6 months of CMM) supports that indicated patients should be treated without delay.

## Supplementary Material

Supplementary data
