# Supplementary material for: Long-term efficacy of high-frequency (10 kHz) spinal cord stimulation for the treatment of painful diabetic neuropathy: 24-Month results of a randomized controlled trial
Source: Diabetes Res Clin Pract. Author manuscript; Available in PMC 2024 Sep 1. (PMC10801706; doi:10.1016/j.diabres.2023.110865)
Supplement: Supplementary data [file NIHMS1957874-supplement-Supplementary_data.docx]

**Neurological Evaluation**

**Motor strength**

Motor strength of the lower limbs was assessed for hip flexion, knee extension, knee flexion, ankle dorsiflexion, and ankle plantarflexion. With the participant in a relaxed, seated position on an exam bench with leg freely hanging above the ground, the clinician administered counterpressure with the hand against the appropriate anatomic location for each assessment to evaluate muscle power during each motion. For each motion, the standard MRC scale was used by the clinician to score the motor strength as one of the following: 0 = No detectable muscle activity, 1 = Palpable or visible contraction without joint movement, 2 = Full range of motion when gravity removed, 3 = Full range of motion against gravity but not against resistance, 4 = Full range of motion against resistance/gravity with less than normal power, or 5 = Normal power.

**Sensory Function**

Sensory function of the lower limbs was assessed via three sensory tests:

1. Light touch testing: The clinician administered light touch at each of the following six dermatomes bilaterally: L1, L2, L3, L4, L5, and S1. With the participant’s eyes closed, the clinician administered light touch with the hand at each dermatome and asked the participant to identify when the touch was felt and if the touch was normal, diminished, or hypersensitive. For each dermatome and side (right and left), the clinician scored the response as one of the following: normal, diminished, absent, or hypersensitive.
2. Monofilament testing: Using the Neuropen device, a 10-g monofilament was used to evaluate the sensation with monofilament touch at 10 points per foot (20 points total) (Figure S1). With the participant’s eyes closed, the clinician applied the end of the monofilament to one point on the foot until the monofilament became bent, indicating a 10-g force being applied. The clinician then asked the participant to identify the sensation as normal, diminished, absent, or hypersensitive. The 10-g force application was conducted for each of the 20 points.
3. Pinprick testing: Using the Neuropen device, a sharp metal pin was used to evaluate the pinprick sensation at 10 points per foot (20 points total) (Figure S1). With the participant’s eyes closed, the clinician applied the pin to one point on the foot until the pin was fully depressed, indicating that a consistent maximum force was applied. The clinician then asked the participant to identify the sensation as normal, diminished, absent, or hypersensitive. The pinprick application was conducted for each of the 20 points.

Figure S1. Locations used for monofilament and pinprick testing. Each neurological exam consisted of testing at 10 points per foot (20 points total), as shown.

**Reflexes**

Reflexes of the lower limbs were assessed via bilateral reflex testing of the patellar tendon (knee), Achilles tendon (ankle), and Babinski reflex (plantar surface of the foot). With the participant in a relaxed, seated position on an exam bench with leg freely hanging above the ground, the clinical assessed each reflex using standard clinical practice. For the patellar and Achilles reflexes, a standard reflex hammer was used to elicit the reflex. For the Babinski reflex, the clinician used the flat side of a pen to contact the plantar surface of the foot. Then, the clinician ran the pen up the side of the foot and across the ball of the foot to evaluate the response. For each of the patellar and Achilles reflexes, the clinician scored the response as one of the following: 0 = Absent/near absent, 1 = Normal, or 2 = Abnormally hyperactive, often with clonus. For each of the Babinski reflexes, the clinician scored the response as one of the following: Abnormal or Normal.

**Criteria for Determining Neurological Change**

For each functional category (sensory function, motor strength, and reflexes), investigators characterized the findings as improved, maintained, or a deficit as compared with baseline status, based on the following prespecified definitions:

- A clinically meaningful neurological improvement was defined as a significant persistent improvement in neurological function that impacts participant’s well-being, is attributable to a neurological finding, and is new or improved as compared with the baseline assessment.
- A clinically meaningful neurological deficit was defined as a treatment-related significant persistent abnormality in neurological function that impacts participant’s well-being, is attributable to a neurological finding, and is new or worsened as compared with the baseline assessment.
- If neither a clinically meaningful neurological improvement nor a clinically meaningful neurological deficit was observed, then neurological status is maintained.

For a clinically meaningful change from baseline, “persistent” was defined as lasting beyond what would be expected for a transient event in this population and unable to be resolved through device reprogramming.

To evaluate overall neurological change at each time point, the following criteria were used to identify each participant as having improvement, maintenance, or deficit:

- Improvement: Improvement in at least one functional category (sensory function, motor strength, or reflexes) without deficit in any functional category (sensory function, motor strength, or reflexes).
- Maintenance: Maintenance in all functional categories (sensory function, motor strength, and reflexes).
- Deficit: Deficit in any functional category (sensory function, motor strength, or reflexes).

**Investigator Training**

Prior to performing any study-related neurological assessments, investigators were trained to conduct the standardized neurological exam. Training for motor strength and reflex testing was conducted via in-person and video training modules developed with two board-certified neurologists.
